# The pathologic and diagnostic in magnetic resonance imaging of brain and cervical spine of patients with neuromyelitis optica spectrum disorder

**Published:** 2018-04-04

**Authors:** Roshanak Mehdipour-Dastjerdi, Fereshteh Ashtari, Vahid Shaygannejad, Marjan Mansourian, Ali Safaei

**Affiliations:** 1 Department of Neurology, School of Medicine, Isfahan University of Medical Sciences, Isfahan, Iran; 2 Isfahan Neuroscience Research Center, Isfahan University of Medical Sciences, Isfahan, Iran

**Keywords:** Devic Disease, Neuromyelitis Optica, Magnetic Resonance Imaging, Demyelinating Disease, Cervical Spine

## Abstract

**Background:** Neuromyelitis optica spectrum disease (NMOSD) is a chronic inflammatory demyelinating disorder that involves central nervous system (CNS) with high affinity for involvement of optic nerve and spinal cord. In current study, due to high prevalence of NMOSD in Isfahan, Iran, we have aimed to assess brain and spine magnetic resonance imaging (MRI) of patients with NMOSD.

**Methods:** This cross-sectional study was performed on 62 patients with diagnosis of NMOSD, who referred to MS clinic of Kashani hospital, Isfahan City, during 2015-17. Patients' age, age of onset, primary brain and spine MRI findings, and expanded disability status scale (EDSS) were recorded in check list. Patients underwent new brain and spine MRI. Data were analyzed with SPSS software. Descriptive data were reported by mean ± standard deviation (SD).

**Results:** 62 known cases of NMOSD including 9 (14.5%) men and 53 (85.5%) women with mean age of 34.32 ± 10.26 years, mean age of onset of 28.03 ± 12.09 years, and mean EDSS of 2.63 ± 1.55 were assessed. 33.9% of patients were anti-neuromyelitis optica (NMO) antibody seropositive. Longitudinal extensive transverse myelitis (LETM) and segmental spinal lesions were found in 66.1% and 29.0% of patients, respectively. Diagnostic NMO brain lesions and posterior periventricular lesions were seen in 38.7% and 67.7% of patients, respectively. Two patients had tumefactive lesions.

**Conclusion:** In current study, we found high rate of posterior periventricular lesions in brain MRI and segmental lesions in spine MRI of both anti-NMO antibody seropositive and seronegative patients. As these lesions are not in NMOSD diagnosis criteria, more considerations are recommended. In addition, tumefactive lesions in patients with NMOSD were an exciting finding of this study that should be discussed about more.

## Introduction

Neuromyelitis optica spectrum disease (NMOSD), also known as Devic disease, is a chronic inflammatory demyelinating disorder that involves central nervous system (CNS) with high affinity to optic nerve and spinal cord involvement.^1^ Since this disorder may lead to optic neuritis (ON) and longitudinal extensive transverse myelitis (LETM), it can pose severe disability in patients.^2^

Regarding the primary signs/symptoms of Devic disease that are similar to those of multiple sclerosis (MS), it was considered as a variant of MS for a long time, and even called Asian MS or Opticospinal MS,^3^ but different clinical, radiological, and immunopathological findings helped differentiation of NMOSD from MS. Thus, it is known as a distinct disorder.^4^

The prevalence of this disorder is estimated to be 1-4.4 persons in 100000 cases and affects women considerably more than men (7.8:1-9:1) (female to male ratio). Mean age of involvement has been reported about 39 years; however, there were reports of NMOSD in children and adults.^3^^,^^5^^,^^6^ NMOSD diagnosis has been made based on 2014 revised criteria designed by Wingerchuk, et al.^7^

Brain magnetic resonance imaging (MRI) inconsistent with MS criteria can help differentiation of these two disorders. In addition, as mentioned above, LETM is considered as one of the basic findings for NMOSD diagnosis. Therefore, complete brain and spinal MRI as a rapid, non-invasive, and available option should be used for first steps of diagnosis.^8^

Primarily it was supposed that brain MRI of patients with NMOSD is either normal or not supported by MS criteria, but recently, some studies have reported involvement of brain stem and diencephalon in NMOSD affected patients.^3^^,^^9^ 51% to 89% of patients with NMOSD have abnormal brain MRI, and even some patients without symptoms have brain lesions which indicate brain involvement as primary expression of NMOSD.^10^ The most remarkable point in Devic patients’ MRI, is involvement of regions with high expression of aquaporin 4, as the most significant marker for differentiation of MS and NMOSD, including hypothalamus, periependymal regions, dorsal and central area of brain stem, corticomedullary junction, and corticospinal tract.^4^^,^^11^

In the current study, due to few studies about brain MRI of patients with NMOSD and also high prevalence of NMOSD in Isfahan City, Iran, we aimed to assess brain and spine MRI of patients with NMOSD.

## Materials and Methods

This cross-sectional study was performed on 62 patients with diagnosis of NMOSD, based on 2014 revised criteria, who referred to MS clinic of Kashani hospital, Isfahan City (a referral center for MS patients affiliated to Isfahan University of Medical Sciences) during 2015-17.

Inclusion criteria were as following: 1) diagnosis of NMOSD or NMOSDSD based on revised criteria of 2014,^7^ 2) presence of records in MS clinic of Kashani hospital, 3) age more than 18 years old, 4) lack of microvascular diseases like diabetes, hypertension, and renal failure, 5) availability of primary brain and spine MRI at the time of diagnosis, 6) no pulse therapy in past three months, 7) availability of primary anti-NMOSD-IgG result at the time of diagnosis.

Patients who did not accept taking new MRI or did not accept rechecking anti-NMOSD antibody and also those who did not refer for follow-up visit to record new results were excluded.

This study was approved based on 395349 codes from Research Council and Ethics Committee of School of Medicine of Isfahan University of Medical Sciences, Isfahan City.

Patient's age, age of onset, primary brain and spine MRI findings, primary anti-NMOSD antibody and expanded disability status scale (EDSS) were recorded in check list. In case of being in acute relapse, EDSS evaluation postponed to remission time. Remission time was defined as one to three months after acute relapse based on severity of symptoms/signs. Moreover, the patients were requested to take new brain and spine MRI and also recheck their anti-NMOSD antibody. 

Anti-NMOSD antibody was checked with cell based method in a single similar laboratory for all patients.

All patients underwent new brain and spine MRI, done with Philips Ingenia 1.5 Tesla MRI device. Imagings were with protocols of T1, T2, and flair for all patients.

Spinal cord lesions were divided to categories of segmental and LETM. 

Brain MRI findings were recorded as well. Brain lesions, included in revised criteria of 2014, were considered as diagnostic lesions.^12^ Then data were analyzed with SPSS software (version 20, IBM Corporation, Armonk, NY, USA). Descriptive data were reported by mean ± standard deviation (SD).

## Results

In the current study, 62 known cases of NMOSD including 9 (14.5%) men and 53 (85.5%) women were evaluated. 

**Table 1:** Findings of spine and brain magnetic resonance imaging (MRI) and symptoms of patients with neuromyelitis optica spectrum disease (NMOSD)

**Pathologic finding**	**Type**	**Patients**	**New findings [n (%)]**
Spinal lesions	LETM	All patients	41 (66.1)
		Anti-NMOSD positive patients	15 (71.4)
	Segmental spinal lesions	All patients	18 (29.0)
		Anti-NMOSD positive patients	4 (19.0)
Brain lesions	Diagnostic lesion	All patients	24 (38.7)
		Anti-NMOSD positive patients	7 (33.3)
	Posterior periventricular lesion	All patients	42 (67.7)
		Anti-NMOSD positive patients	12 (57.1)
	Tumefactive lesion	All patients	2 (3.2)
		Anti-NMOSD positive patients	0 (0)
Symptoms	ON	All patients	36 (58.1)
		Anti-NMOSD positive patients	14 (66.7)
	TM	All patients	24 (38.7)
		Anti-NMOSD positive patients	7 (33.3)

NMOSD: Neuromyelitis optica spectrum disease; LETM: Longitudinal extensive transverse myelitis; ON: Optic neuritis; TM: Transverse myelitis

Mean age of participants was 34.32 ± 10.26 years. Mean age of onset in all patients was 28.03 ± 12.09. Mean EDSS of all patients was 2.63 ± 1.55.

Anti-NMOSD antibody was assessed in patients, and results showed 21 (33.9%) positive patients. Nineteen (90.5%) of them were women and two (9.5%) were males. Mean age of onset in anti-NMOSD IgG positive patients was 28.19 ± 12.13. EDSS among anti-NMOSD positive patients was 2.47 ± 1.49.

Brain and spine MRI of patients at the time of diagnosis were evaluated as well. 27 (43.5%) patients had LETM that 10 (47.6%) of them were anti-NMOSD positive patients. 

17 (27.4%) patients showed brain lesions, and 6 (28.6%) of them were anti-NMOSD positive. These brain lesions were in the following areas: 3rd and 4th ventricular periependymal area, medulla, area postrema, pons, midbrain, thalamus, hypothalamus, and diencephalon.

ON was the first presentation of 36 (58.1%) patients, and 24 (38.7%) patients presented transverse myelitis (TM) as the first symptom. Two remained patients presented vertigo, nausea, and ataxia as their first presentation.

41 patients (66.1%) showed both ON and TM during their disease regardless of considering their first presentation of disease.

In seropositive group, 14 (66.7%) patients reported ON, and 7 (33.3%) patients presented TM as their first symptom of NMOSD. Among anti-NMOSD antibody positive patients, 12 (57.1%) patients showed both ON and TM in duration of their disease regardless of considering first presentation. Findings of spine and brain MRI as well as symptoms of patients are shown in table 1.

Types of brain lesions in patients regarding anti-NMO antibody seropositivity are shown in table 2. The most prevalent lesion was periependymal area of 3^rd^ and 4^th^ Ventricles.

**Table 2:** Brain lesions of studied population

**Patients**	**Anti-NMO Ab Positive (%)**	**All (%)**
Thalamus	14.28	12.90
Hypothalamus	9.52	9.67
Periepandymal Area of 3^rd^ and 4^th^ Ventricles	28.57	25.80
Midbrain	4.76	8.06
Pons	14.28	16.12
Medulla	9.52	11.29
Area Postrema	9.52	9.67

NMO: Neuromyelitis optica; Ab: Antibody

**Figure 1:**
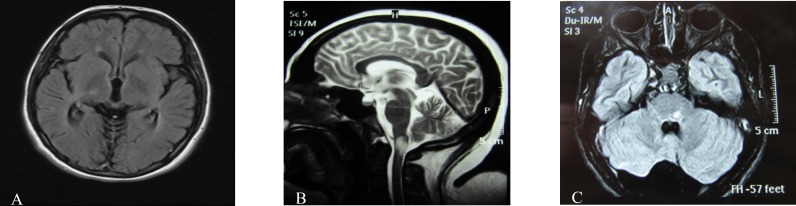
Specific brain lesions: Right Thalamus, area postrema, brain stem (left to right)

## Discussion

NMOSD is almost an unknown disorder and neuro-imaging information about this disorder is limited. We have evaluated neuro-imaging findings, anti-NMO IgG, and symptoms of patients with NMOSD.

This study was conducted on 62 known cases of patients with NMOSD in Isfahan City. One of the advantages of current study is larger population of this study in comparison to previous ones which had 20-40 patients.^9^^,^^13^

Mean age of onset in current study was lower than that of previous studies which was related to 3rd to 4th decades,^14^^,^^15^ but gender distribution is similar to previous studies, as other reports presented 66%-88% women involvement in NMOSD.^16^

Identification of anti-NMO IgG or anti-aquaporin 4 channel, as a diagnostic marker for NMOSD with 91% specificity, opened new windows in diagnosis of Devic disease, and also differentiation of this disorder from MS.^17^ We found this antibody in 33.9% of our patients that is higher than previous studies conducted in this area.^13^ This antibody is hypothesized to be in accordance with higher prevalence of relapses and worse prognosis,^18^ while there is another study that presented no association.^19^

This antibody showed role of channelopathy as well as abnormal humoral immune system in NMOSD. Another point that should be considered is about neuro-imaging of this disorder. By considering anti-NMO antibody as a specific marker of NMOSD, brain regions with high expression of aquaporin 4 should be sought.^20^ It is an important point which is mentioned in the study of Marignier, et al. that found brain lesions in aquaporine-4 high expression areas including; periaqueductal grey matter, hypothalamic area, and area postrema. These findings were seen in anti-NMO seropositive patients.^21^ Wingerchuk, et al. added these areas to 2014 diagnostic criteria of NMOSD.^12^

In this study, 27.4% of all patients had brain lesions at the time of diagnosis. Brain involvement is different in Western and Eastern communities; eastern ones have reported up to 60% of brain involvement while in west it was less than 30% which is consistent with our study.^2^ However, existence of brain lesion at the time of diagnosis among anti-NMO positive patients was 28.6% which is not considerably higher than seronegative ones. Moreover, this lower percentage of brain lesions finding in primary MRI may be due to difference of devices, as some of these images were from previous decades and were taken by old devices with low-resolution. 

The lesions in last brain MRI of patients were as follows: 24 (38.7%) patients had diagnostic lesions based on revised criteria,^12^ and seven patients were seropositive. This low rate is somewhat a paradox that may neglect the role of aquaporin 4 channelopathy or maybe due to incorrect laboratory findings. As type and number of brain lesions may change over time, brain MRI follow-up is suggested (Figure 1).^20^

**Figure 2:**
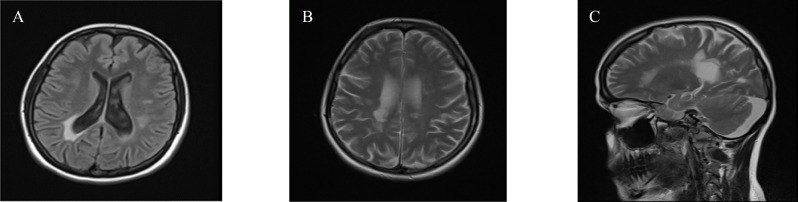
Posterior periventricular lesions

Another important finding in brain MRI was posterior periventricular lesions found in 67.7% of all patients, and also 57.1% of anti-NMO IgG positive patients (Figure 2).

This area has not been considered as a diagnostic area for NMOSD.^12^ but we have found remarkable prevalence of it in our patients and also in seropositive ones. Thus, further studies by considering this area in patients with Devic disease is recommended. 

Another remarkable point is existence of tumefactive lesions in 2 of our patients. Despite necrotizing basis of NMOSD in two patients, we found tumefactive lesions as inflammatory lesions that can be seen in MS.

**Figure 3:**
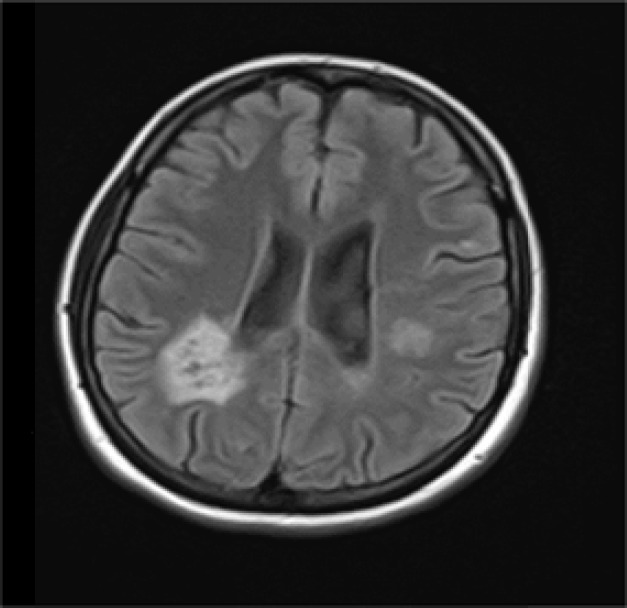
Tumefactive lesion

This type of lesion was reported in another case-report as well.^22^ Therefore, we suggest NMOSD as a differential diagnosis in case of finding tumefactive lesions (Figure 3).

**Figure 4:**
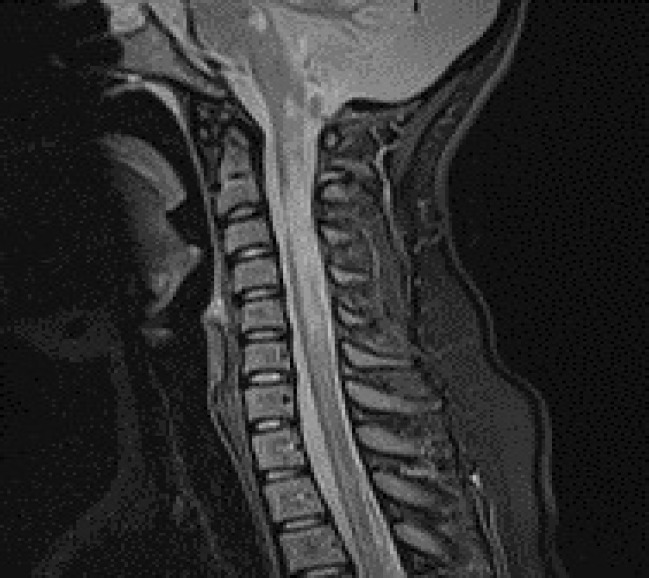
Longitudinal extensive transverse myelitis (LETM)

LETM is a diagnostic criterion in NMOSD. It was found in less than half of patients at the time of diagnosis, and in new assessment it was found in 66.1% of them. Ashtari, et al.^13^ mentioned similar results (Figure 4).

**Figure 5:**
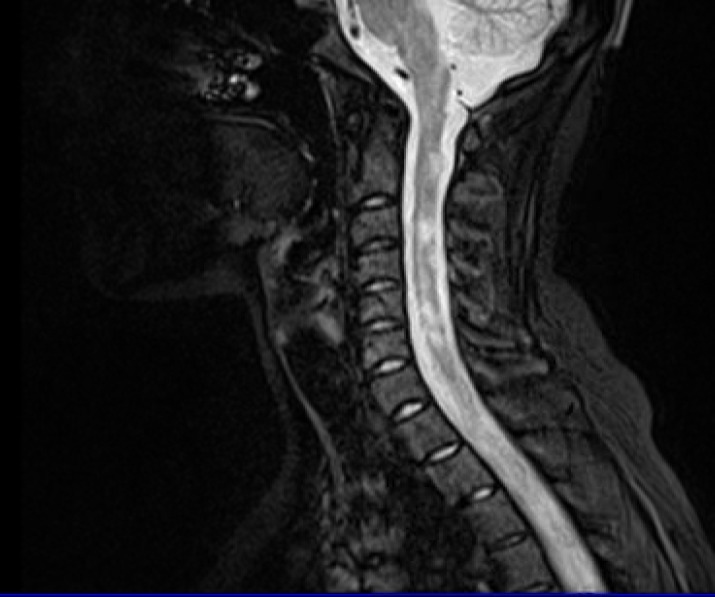
Cervical segmental lesions

The important point is finding of segmental lesions that is not considered diagnostic but is found in about 30% of our patients (Figure 5). This finding is notably higher in comparison to Liao, et al. study.^2^

ON and TM are usually the first and also characteristic symptoms of NMOSD as the current study reports. Previous studies have mentioned that ON/TM presentation as first symptoms are more common in anti-NMO seropositive patients. This is similar to the present study in that 57.1% of seropositive patients mentioned ON/TM as the first presentation of their disease. Moreover, they have reported that simultaneous occurrence of ON and TM and also bilateral ON are more usual in seronegative patients,^14^ which is not assessed in our patients. Another point that should be mentioned is occurrence of vertigo and ataxia in two of patients as first presentations of NMOSD, which shows probable brain lesions in regions with high expression of aquaporin 4 channels.

Since cross-sectional studies may have assessment limitations and also NMOSD signs, symptoms and imaging findings overlap with those of MS, cohort studies for better evaluation and thorough assessment of these patients are recommended to complete more comprehensive criteria for diagnosis of NMOSD and its differentiation from MS.

## Conclusion

In current study we found high rate of posterior periventricular lesions in brain MRI and segmental lesions in spine MRI of both seropositive and seronegative anti-NMO antibody patients. As these lesions are not in NMOSD diagnosis criteria, more considerations are recommended. In addition, tumefactive lesion in patients with NMOSD was an exciting finding of this study that should be discussed about more.

## Notes:

**How to cite this article:** Mehdipour-Dastjerdi R, Ashtari F, Shaygannejad V, Mansourian M, Safaei A. The pathologic and diagnostic in magnetic resonance imaging of brain and cervical spine of patients with neuromyelitis optica spectrum disorder. Iran J Neurol 2018; 17(2): 58-63.
